# Techniques for *In Vivo* Measurement of Ligament and Tendon Strain: A Review

**DOI:** 10.1007/s10439-020-02635-5

**Published:** 2020-10-06

**Authors:** Qiang Zhang, Naomi C. Adam, S. H. Hosseini Nasab, William R. Taylor, Colin R. Smith

**Affiliations:** Institute for Biomechanics, ETH Zürich, Leopold-Ruzicka-Weg 4, 8093 Zürich, Switzerland

**Keywords:** Ligament strains, Tendon strains, Biosensors, Loading conditions, Soft tissue function, *In vivo* measurement, Dynamic imaging, Stretchable sensors

## Abstract

The critical clinical and scientific insights achieved through knowledge of *in vivo* musculoskeletal soft tissue strains has motivated the development of relevant measurement techniques. This review provides a comprehensive summary of the key findings, limitations, and clinical impacts of these techniques to quantify musculoskeletal soft tissue strains during dynamic movements. Current technologies generally leverage three techniques to quantify *in vivo* strain patterns, including implantable strain sensors, virtual fibre elongation, and ultrasound. (1) Implantable strain sensors enable direct measurements of tissue strains with high accuracy and minimal artefact, but are highly invasive and current designs are not clinically viable. (2) The virtual fibre elongation method tracks the relative displacement of tissue attachments to measure strains in both deep and superficial tissues. However, the associated imaging techniques often require exposure to radiation, limit the activities that can be performed, and only quantify bone-to-bone tissue strains. (3) Ultrasound methods enable safe and non-invasive imaging of soft tissue deformation. However, ultrasound can only image superficial tissues, and measurements are confounded by out-of-plane tissue motion. Finally, all *in vivo* strain measurement methods are limited in their ability to establish the slack length of musculoskeletal soft tissue structures. Despite the many challenges and limitations of these measurement techniques, knowledge of *in vivo* soft tissue strain has led to improved clinical treatments for many musculoskeletal pathologies including anterior cruciate ligament reconstruction, Achilles tendon repair, and total knee replacement. This review provides a comprehensive understanding of these measurement techniques and identifies the key features of *in vivo* strain measurement that can facilitate innovative personalized sports medicine treatment.

## Introduction

In addition to considerable pain and loss of function experienced by patients,^4^ musculoskeletal soft tissue injuries present a considerable financial burden to health care systems worldwide. Some 200’000 anterior cruciate ligament (ACL) injuries^76^,^109^ occur annually in the Unites States alone, and nearly half of them require reconstructive surgery.^37^ The socioeconomic costs of the resulting medical treatment have been estimated to be over one billion dollars annually.^58^,^123^ In Sweden, the average cost of surgical management of acute Achilles tendon (AT) ruptures in 2009-2010 was around $10,000.^178^ Moreover, revision rates following initial treatment for AT rupture (2%-8%)^89^,^130^ and ACL revisions (3-16%)^122^,^176^ suggest that more effective treatment offer considerable potential exists for not only reducing costs, but also improving patients’ functional outcome.

Transforming sports medicine therapies to address current treatment shortcomings requires a comprehensive understanding regarding the functional demands withstood by musculoskeletal soft tissues during activities of daily living in healthy, injury, and pathologic conditions. A detailed understanding of *in vivo* ligament and tendon strains is not only important for identifying injury mechanisms, informing surgical reconstructions and optimizing rehabilitation protocols, but also essential for defining physiologic loading conditions for *in vitro* experiments and tissue engineered constructs. Therefore, the measurement of *in vivo* musculoskeletal soft tissue strain patterns during functional movements is critical for both facilitating scientific discoveries and driving innovative clinical treatments.

The importance of soft tissue strain to biomechanical tissue function and adaptation has been well established. In 1847, Wertheim demonstrated that the stress-strain relationship in animal tissue does not follow the linear relationship dictated by Hooke’s law.^177^ In the 1960s, the introduction of Fung’s Law provided the contemporary understanding of soft tissue viscoelasticity, building a more complete concept of the tissue deformation response to loading.^53^ Shortly afterwards, it was discovered that both soft tissue strain magnitude and rate had a direct influence on the failure properties of ligaments^124^ and tendons.^61^ In 1992, it was discovered that the strain magnitude, not stress, experienced by muscle fibres was directly related to damage severity.^51^ However, despite the importance of soft tissue strain being recognized for decades, clinical translation of this knowledge has been hindered by the limited *in vivo* measurements of tissue strain magnitudes and rates during dynamic movements.

In clinical settings, improved knowledge of *in vivo* ligament strains^67^ has facilitated key innovations in reconstruction techniques and rehabilitation for ligament injuries.^92^ For example, in the 1990s, it was thought that the bone tunnel during ACL reconstruction surgeries should be positioned to achieve graft isometry throughout flexion.^6^,^190^ However, research into the role of loading on graft tissue health^25^,^29^ and the contribution of ACL strain to knee stability^179^ led to an evolution of the surgical technique such that current approaches aim to position the tunnels in the centre of the native ACL attachment footprints to better replicate the strain patterns experienced by the native tissue.^141^ With respect to rehabilitation, sensor measurements of *in vivo* ACL strain showed a significant increase when the knee moved to extended postures,^20^,^62^ which has informed activity selection for post-operative rehabilitation. However, innovation of clinical therapies for many other pathologies is hindered by the limited *in vivo* strain data available for many other ligaments and tendons, especially during functional and rehabilitative activities.

*In vivo* strain measurements also provide critical insights into muscle-tendon function by enabling the elongation of the muscle fibres, tendon, and aponeurosis to be independently quantified.^33^ Measurements of muscle fibre lengths and velocities during movement enable investigation into how individuals leverage optimal force-length and force-velocity muscle contraction conditions. Ultrasound studies indicate that the tendon can act as a buffer to overall muscle-tendon unit stretch, resulting in strains within the individual tissues that do not reflect the overall length change of the muscle-tendon unit.^33^ For example, during early stance in walking, the overall length of the triceps surae muscle-tendon unit increases substantially, but the lateral gastrocnemius fibres lengthen only to a small degree,^115^ while the medial gastrocnemius and soleus fibres even remain isometric.^3^ At push-off, all of the triceps surae muscles shorten, but at a much slower rate than the tendon and muscle-tendon unit as a whole. This demonstrates the importance of *in vivo* strain measurements to reveal the complex interactions within muscle-tendon units to conserve energy during muscle contraction, amplify power output, and absorb shocks during impact.^142^

An improved understanding of *in vivo* soft tissue strains will also provide important insights into how humans modulate their neuromuscular coordination to generate movement. When coupled with traditional motion analysis techniques, *in vivo* strain measurements could enable an improved understanding of how the body leverages elastic energy storage in tendons.^191^ Furthermore, dynamic strain measurements of all the soft tissues crossing a joint may provide key insights towards resolving muscle redundancy.^63^ This problem seeks to determine how the externally measurable joint torques during movement are distributed to the redundant musculoskeletal system, resulting in one of the most famous unsolved problems in biomechanics.^137^

The critical need for *in vivo* quantification of musculoskeletal tissue strain for both clinical and basic science applications has inspired the development of three main measurement techniques: (1) implantable strain sensors, (2) virtual fibre elongation, and (3) ultrasound. Implantable strain sensors have largely been applied to measure *in vivo* ACL strains,^17^,^44^,^140^ but such technologies have mostly been implemented during controlled movements and in sterile surgical environments. The virtual fibre elongation method uses bone position from image-based methods including fluoroscopy,^87^,^166^,^181^ ultrasound,^134^ open magnetic resonance imaging (open-MRI),^64^,^91^ or computed tomography (CT)^60^,^161^ to derive *in vivo* ligament and tendon strains. Ultrasound technique enables dynamic non-invasive planar imaging of soft tissue structures, and has mostly been applied to measure muscle^33^,^105^ and tendon^126^,^159^ deformation. Each technique presents different advantages and limitations regarding accuracy, invasiveness, safety, and activities that can be measured. In addition, previous studies have chosen different approaches to define the fibre reference length.^19^,^69^,^71^,^75^ As a result, the reported strain data may not represent the real soft tissue strain, and thus understanding the true loading condition within the tissue remains challenging. Therefore, the abilities of these measurement techniques to determine the slack length of soft tissue should be fully reviewed and discussed. The technologies involved in *in vivo* strain measurement have been previously reviewed,^16^,^44^,^140^,^144^ however there has not yet been a comprehensive summary of the key findings and clinical impacts of each methodology. The purpose of this study is therefore to provide an overview of *in vivo* tendon and ligament strain measurement methodologies and their key findings, clinical implications, and limitations, as well as to identify technological developments that may lead to clinical and basic science advances in sports medicine.

## Materials and Methods

### Literature Search and Selection

Articles were searched using the keywords “sensor*”, “gauge*”, and “transduc*”, “imag*”, “ultrasound*”, “fluoroscop*” as well as “tendon*”, “ligament*, “muscle*”, “length change*”, “strain*” and “elongat*” in the Pubmed and EMBASE databases. A total of 9842 articles were found. The titles and abstracts of all articles were firstly screened by one author to exclude clearly irrelevant articles. Two authors then reviewed the full texts of the remaining articles to identify articles discussed in this review. As this is a narrative review, the two authors also searched the reference lists of these articles to identify any other relevant articles that had not been found through the above search. One experienced author discussed these candidate articles with the two authors to make a final decision on their inclusion. Finally, 151 articles were includedor reviewing.

### Strain Calculation

Strain (*ε*) is traditionally defined using Eq. (1) in the biomechanics literature:

1 $$\varepsilon = \frac{{l - l_{0} }}{{l_{0} }}$$

where $l$ is the tissue length and $l_{0}$ is the slack length or reference length. Slack length is the length at which a tissue begins exhibiting force when it is stretched. The ability of each measurement technique to quantify tissue slack length is detailed in the following sections. In many studies, the true slack length was not measured and instead a reference length, the length of the tissue in a defined body posture is used to calculate strain.^71^,^108^ Thus, comparing strains between studies requires careful consideration of the $l_{0}$. definition.

## Implantable Strain Sensors

Implantable sensors have been employed to directly measure *in vivo* ligament strain patterns in humans. Similar sensors have been applied to measure human tendon forces. Typically, these sensors leverage similar working principles, where mechanical loading or deformation of the tissue induces changes in their electric signals (voltage, resistance, or capacitance). Buckle transducers, liquid metal strain gauges, fibre optic transducers, and sonomicrometry crystals have all been used for *in vivo* force and strain measurements in animal studies to assess muscle, tendon, and ligament function during dynamic activities.^16^,^44^ For example, liquid metal strain gauges have been applied to measure *strain* patterns in rabbit ATs and in horse suspensory ligaments.^26^,^84^ In addition, buckle transducers and fibre optic transducers have been applied to measure AT *force* in humans during walking and jumping.^43^,^52^

While many aforementioned sensor designs have been demonstrated for force measurements in humans and strain measurements in animals, only Hall Effect Strain Transducers (HESTs) and Differential Variable Reluctance Transducers (DVRTs) have been used to directly measure *in vivo* strains in human musculoskeletal tissue.^44^ Therefore, the following discussion focuses on the properties, applications, and limitations of these sensors.

### Measurement Technology and Experimental Methods

**Figure 1:**
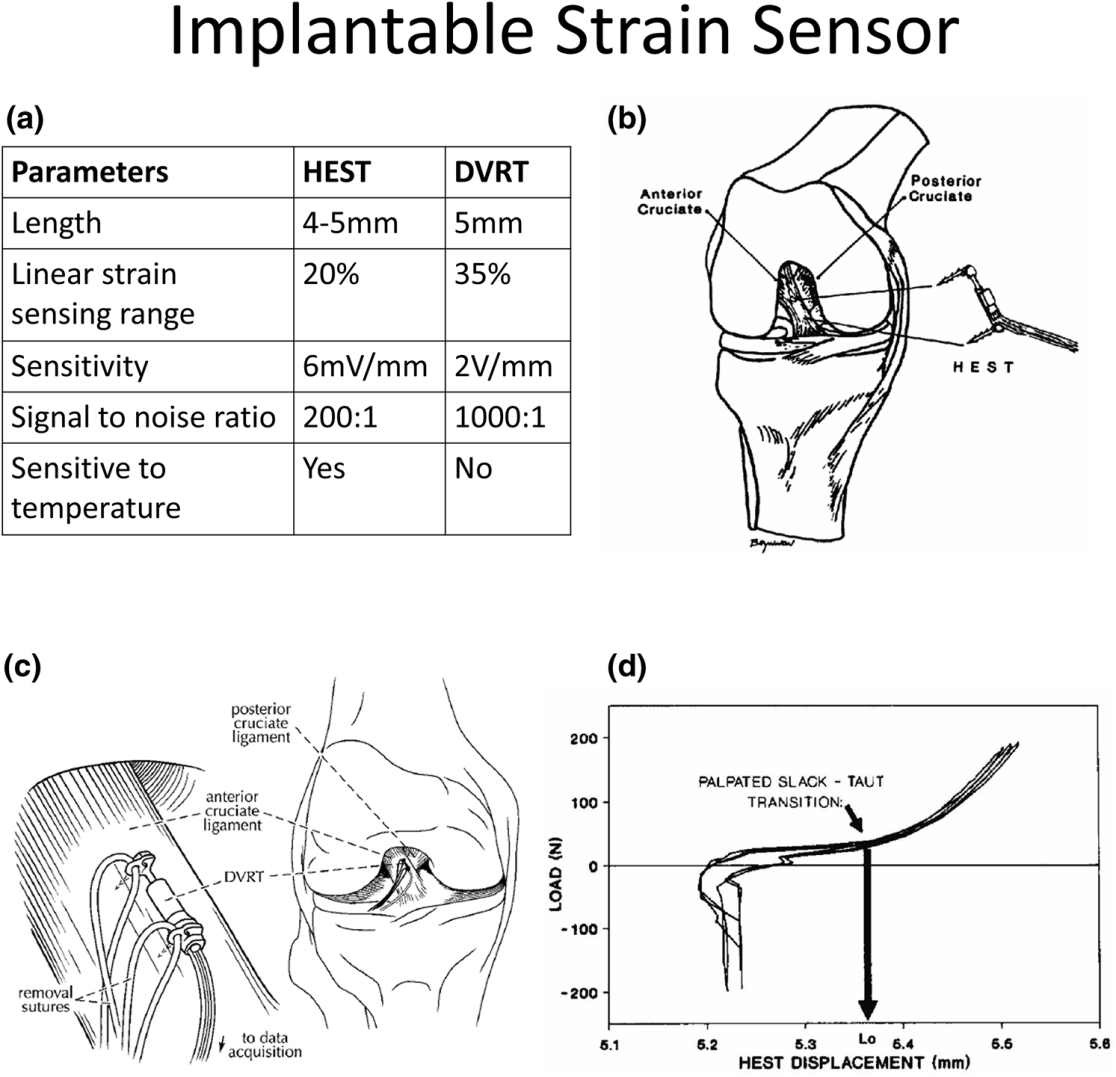
HEST implantation and reference length identification. (a) Parameters of HEST and DVRT sensors. (b) Scheme showing a HEST sensor mounted onto the anteromedial bundle of the ACL with two fixation barbs. (c) Scheme of DVRT sensor fixation. (d) Plot of tibia shear loads against HEST displacement, ACL reference length is defined as the HEST length at the slack-taut transition point. (Included figures are adapted from Ref. 21, 45, and 46).

To detect displacement, HEST sensors measure the change in voltage resulting from relative movement between a magnet and a Hall-effect magnetic sensor, whereas DVRTs measure the movement of a magnetic core within two coil windings by measuring the resulting change in magnetic reluctance.^17^ The parameters of these two sensors are displayed in Fig. 1a.^17^,^44^ Beynnon and co-workers arthroscopically implanted a HEST sensor onto the ACL of subjects under local anaesthesia,^21^ mounting the sensor by pressing on two fixation barbs (Fig. 1b). ACL strains were then quantified during limited functional movements before removal of the sensor. The measurement procedures of DVRT sensors are similar to the HEST sensors (Fig. 1c).^46^

### Slack Length

To assess *in vivo* ACL slack length, Beynnon and co-workers recorded the displacement of a HEST sensor fixed on the ACL while applying antero-posterior shear loads to the tibia.^19^,^45^ They found an “inflection point” on the plot of the sensor length against shear load (Fig. 1d), corresponding to the length where the ACL engages. The corresponding sensor length at this point was considered to be the reference length used to calculate strain. Hence, any subsequent relative change of the sensor displacement from this length was considered to directly reflect loading within the ACL.^19^,^72^ This method was later validated by Fleming and co-workers through concurrently implementing a force probe to measure ACL load on cadaveric knees.^47^

### Key findings

*In vivo* strain sensor measurements have provided critical insights into rehabilitation protocols for ACL reconstruction by providing direct evidence for activity selection that ensure tissue strains are under damaging magnitudes,^62^ but high enough to facilitate healing.^136^ Specifically, squatting was long thought to be a safe activity for early rehabilitation of ACL reconstruction. However, *in vivo* ACL strain measurements using DVRT sensors have revealed that peak strains induced by squatting (3.6%)^20^ are higher than lunges (1.8-2.0%), sit-to-stand (2.8%), step-up/down (2.5-2.6%),^62^ and cycling activities (1.7%).^46^ These findings indicate that squatting exposes the ACL to greater loads than open-chain movements^17^ and should therefore be used cautiously during rehabilitation. *In vivo* sensor measurements have also revealed key insights into the role of muscle activation in both loading and protecting the ACL. The measurements indicate that isometric quadriceps contractions increase ACL strain at flexion angles of less than 50°,^18^,^19^,^72^ isometric gastrocnemius contractions increase ACL strain over all tested flexion angles, and that hamstrings contraction reduce ACL strain at flexion angles greater than 5°.^17^,^48^ These measurements have provided fundamental knowledge that can inform targeted muscle strengthening and neuromuscular coordination training to protect the ACL.

### Advantages and Limitations

There are several unique advantages to the HEST and DVRT implantable sensor approaches for strain measurement. Firstly, it provides a direct strain measurement and thus the data is highly representative of the local tissue strain environments. Secondly, both HESTs and DVRTs are highly sensitive and able to detect minute strains in the musculoskeletal tissues.^17^ Finally, sensors provide high sampling frequencies for strain and strain rate measurements during dynamic activities.

Despite the insightful findings, however, the HEST and DVRT sensors have several major limitations:

1. Invasive nature: Sensor implantation is a highly invasive procedure, resulting in ethical difficulties to justify their use in healthy subjects. For ACL strain measurements, implantations have therefore typically been undertaken together with a pre-planned arthroscopic surgery to perform minor repairs of neighbouring tissues.^19^ Consequently, such patients may have deformities or injuries that may alter the tibiofemoral contact mechanics^99^ and potentially alter the measured soft tissue strain patterns.^28^,^193^
2. Tissue impingement: Current sensors can only be implanted in locations where there is sufficient room for the sensor and straight-forward surgical assess to the tissue. For example, the strain sensors have always been attached to the anteromedial bundle of the ACL because it is more accessible from the anterolateral surgical portal than the posterolateral bundle of the ACL. While HEST and DVRT sensors are small in size, their solid constitution can result in impingement with surrounding musculoskeletal tissues, and therefore impede certain postures during testing. Importantly, sensor disturbance with the femoral notch is unavoidable at full extension of the knee during ACL strain measurements,^44^ which hinders the assessment of ACL strain patterns throughout activities involving full extension or even hyperextension of the knee.^44^
3. Limited implantation duration: Previous experiments have been limited to a few hours because both HEST and DVRT sensors require a cable that crosses the skin to transmit the measurement signal, and are therefore not long-term biocompatible. These limitations have prevented subjects from performing many functional activities of daily living.
4. Sensor-body interference: Sensor cable migration induced by subject body movement can cause significant artefacts in the sensor signal. Sensor cables should be stabilized and well protected during experiments.
5. Sensor implantation alignment: Measurements are sensitive to implantation orientation of the sensor relative to the tissue. To assess tensile ligament strains, the measurement axis of the sensor must be aligned with the ligament fibres during implantation in order to record uniaxial strain.

### Future Work

**Figure 2:**
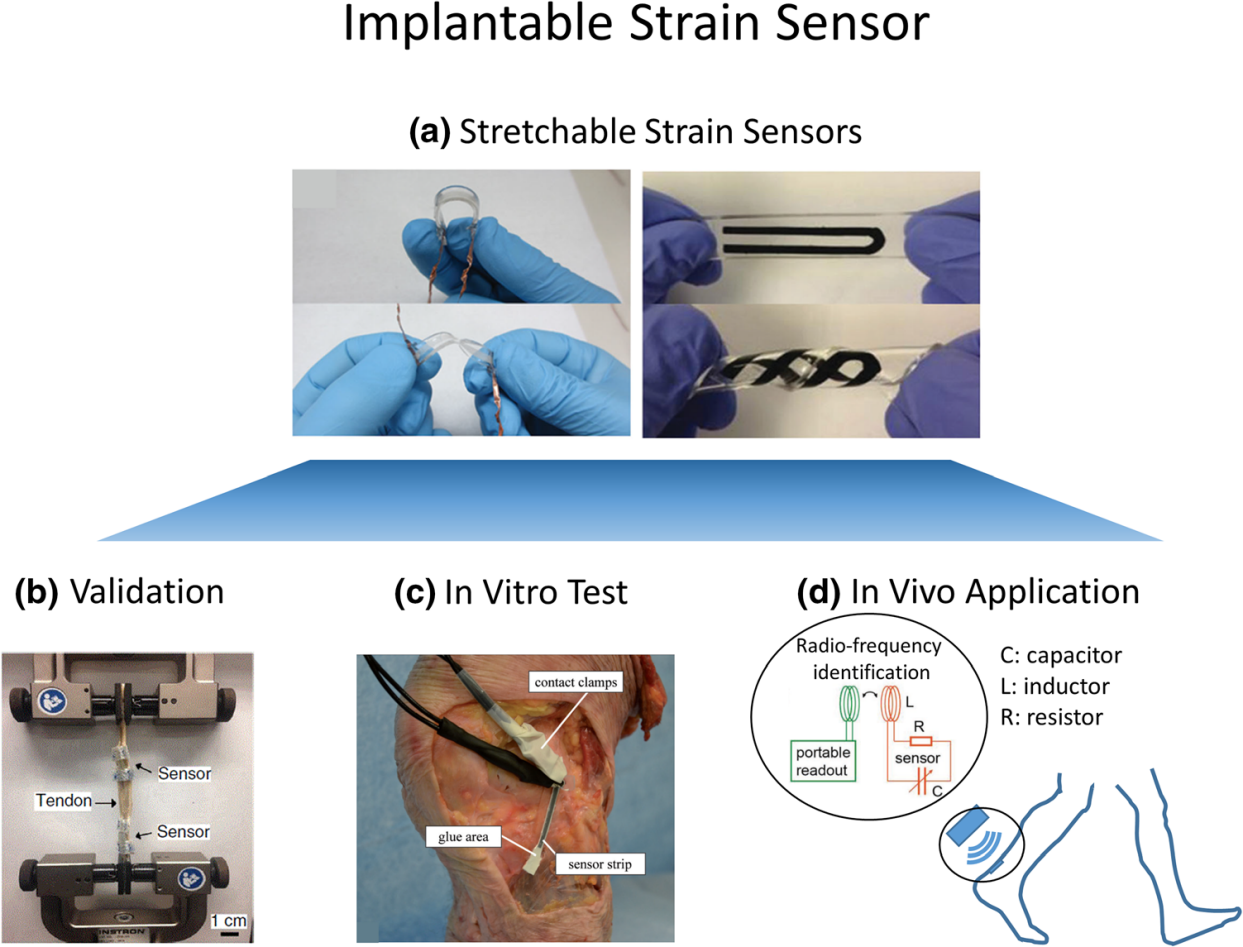
Innovative stretchable strain sensors show great potential for quantifying in vivo musculoskeletal soft tissue strains. (a) Stretchable strain sensors based on soft elastomers and nanomaterials could minimize the risk of interfering with neighbouring tissues. (b) Stretchable sensors have demonstrated high accuracy in measuring tendon strains during uniaxial tensile loading. (c) Stretchable strain sensors have been attached to the anterolateral ligaments using glue to measure ligament strains during knee joint manipulation. d) In vivo wireless measurements using stretchable sensors are possible using radio-frequency identification technology. Here, the passive sensor works as a LCR circuit whose resonance frequency varies with strain and can be readout using inductive coupling. (Included figures are adapted from Refs. 8, 24, 182, and 192).

Recent advances in stretchable electronics may enable clinical translation of strain sensors towards intraoperative guidance and long-term assessment of healing. To facilitate these clinical applications and minimize risk to research subjects, these next generation strain sensors should incorporate three critical features: flexibility, wireless data transmission, and long-term biocompatibility. Thin and soft membrane-like strain sensors can be fabricated by embedding conductive materials in stretchable materials to reduce the risk of tissue impingement.^7^ Initial measurements include skin surface assessments of joint rotation 186 and muscle movement of the trachea,^77^ as well as ex vivo strains in the anterolateral ligament of a cadaveric knee (Fig. 2).^192^ Flexible electronic sensors can be fabricated using biocompatible materials (PDMS, gold, titanium, etc.), and several have demonstrated good biocompatibility through histologic examination.^106^,^174^ Wireless signal transmission with a passive sensor (i.e. no battery or chips) can be achieved using radio-frequency identification technology,^73^ enabling a resonant strain sensor containing a stretchable capacitor and a coil inductor whose resonance frequency can be wirelessly measured by a readout system to be developed (Fig. 2).^158^ However, improvement of sensor fixation,^19^,^143^ as well as long-term stability and quality of flexible sensor signals are still necessary for clinical application. The development of biodegradable strain sensors that are resorbed after a given duration is critical to avoid a second surgery for sensor removal.^24^ Such sensors would be ideal for clinical translation into soft tissue repair surgeries, especially for long-term assessment of tissue healing and data driven physical-therapy.

## Virtual Fibre Elongation

Initial imaging assessments of ligament strain were achieved using open-MRI^9^,^64^ and CT scans^55^,^85^,^138^,^188^ to measure ligament length on the 2D slice images that best displayed the structure. However, substantial inaccuracies were clearly present in such 2D measurements due to out-of-plane errors. The virtual fibre elongation method overcomes this limitation by leveraging imaging data to reconstruct the relative 3D poses of the bones to determine the relative displacements of soft tissue attachment sites to non-invasively quantify tendon and ligament length change patterns.^82^,^83^,^183^ For investigating dynamic activities, fluoroscopy is the state-of-art imaging modality, as CT and MRI are limited by their long-capture times and small field of view (FOV). Thus the following discussion will primarily review fluoroscopic imaging studies.^67^

### Measurement Technology and Experimental Methods

**Figure 3:**
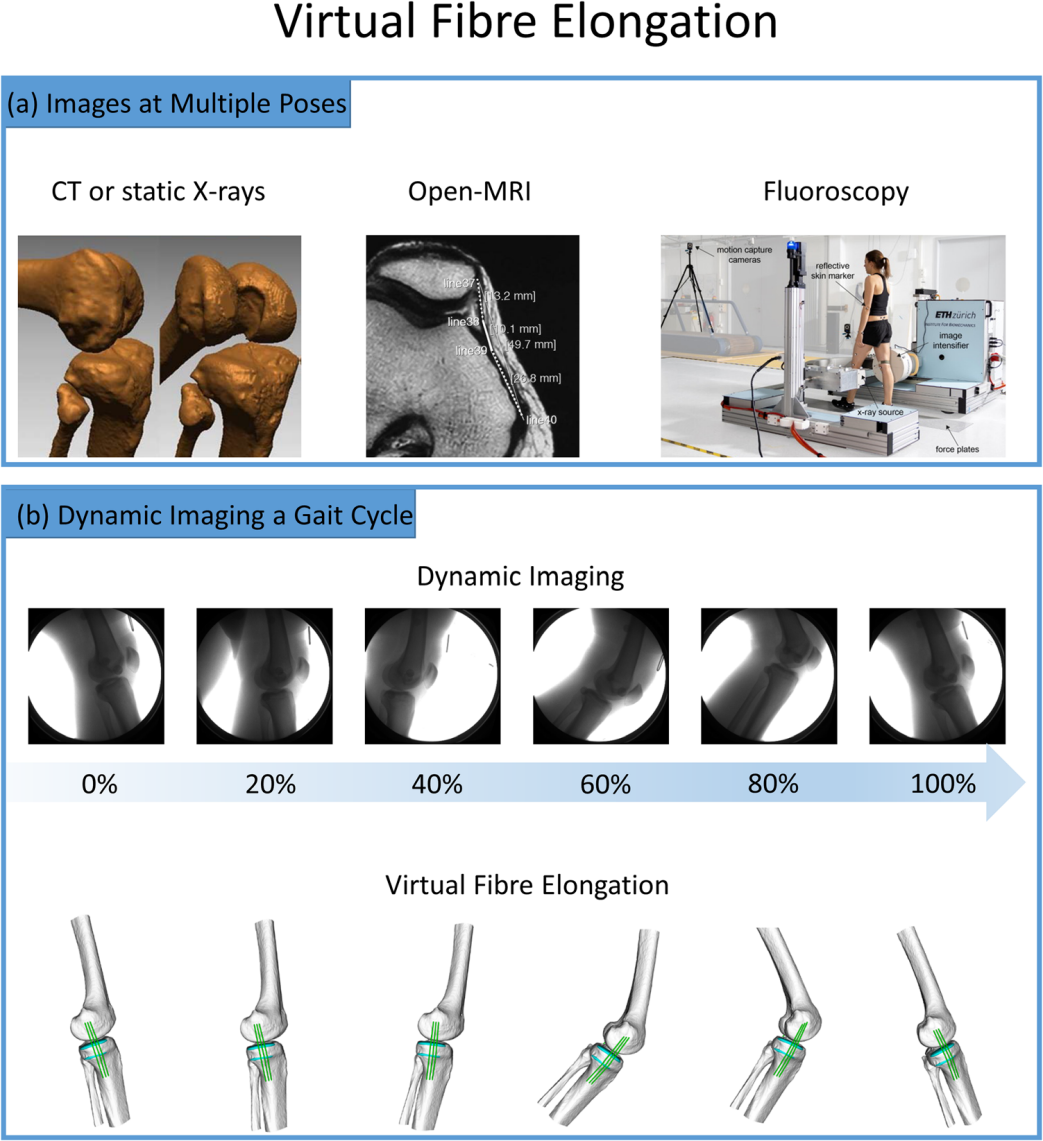
Different virtual fibre elongation methods to quantify in vivo soft tissue strains. (a) Static imaging at multiple poses or dynamic imaging throughout a movement. (b) In this example, dynamic video fluoroscopy is used to measure superficial medial collateral ligament (sMCL) strains during walking. The fluoroscope can move vertically, and the whole system is fixed on a frame that can slide along the rail. A time series of radiographic images of the knee joint are taken. The bone models are matched to the joint postures in each image to quantify the joint kinematics during the gait cycle. sMCL virtual fibres that connect the femoral and tibial attachment sites and wrap the bone surfaces are created. The lengths of the fibres are measured to characterize the long change patterns of the sMCL. (Included figures are adapted from Ref. 64 and 188).

Typically, the joint of interest is statically imaged using MR^171^ or CT,^161^ then segmentation is performed to construct 3D surface models of the bones. The attachment footprints of the ligament bundles are identified on the bone models from the images (MR) or relative to landmarks (CT). Subsequently, the subject performs a functional movement while the joint is imaged using single or dual-plane fluoroscopes^152^,^161^ to acquire a time series of radiographic images of the bones. The 3D bone poses are calculated to match to the images using a manual or (semi-)automated 2D-3D registration software.^27^,^57^,^167^ This process is repeated for each radiographic image to quantify the joint kinematics throughout the movement (Fig. 3).

The length change of a ligament during a movement is assessed by tracking the relative displacements of the attachment points. For ligaments with small attachment footprints, a virtual fibre connecting the centroids of ligament insertion sites is defined as the ligament “longitudinal axis”.^35^ For larger attachments, multiple virtual fibres can be defined.^131^,^173^ The fibre path can either be defined as the straight line connection between attachment points,^131^ or wrapping surfaces can be defined to represent the real curvilinear path of the ligament and prevent penetration of the bone geometries.^71^ The length of the virtual fibre at each joint pose is compared to quantify the relative length change throughout the activity. As the virtual fibre is a geometric path, the orientation of the ligament force can also be quantified as the angle between the projection of the virtual fibre in each plane and the relevant axes in the predefined coordinate system of the joint.^181^,^187^ This enables insights into ligament function such as the restraint it can provide against external loads and graft bending in ligament reconstruction.^162^,^163^

### Slack Length

The virtual fibre elongation method does not provide any inherent means to determine the slack length of a tissue. Accordingly, most studies using this method define the reference length as the length of a tissue in a reference joint posture, to normalize length change measurements and provide an estimate of the elongation patterns. For the knee ligaments, the length at full knee extension^71^,^166^ or heel strike of walking^108^ is commonly used as the reference length.

### Key Findings

The primary advantage of the virtual fibre elongation method is that it enables the non-invasive assessment of multiple ligaments in a joint during dynamic movements. The resulting comprehensive understanding of ligament contributions to functional joint mechanics has informed many surgical and rehabilitative sports medicine procedures. Using the virtual fibre elongation method, ACL length was shown to decrease by 28% from full knee extension to 135° of knee flexion during single leg lunge,^87^ while PCL length increases by 35%.^131^ Ankle plantarflexion and supination were found to increase the length of the anterior talofibular ligament but decrease the length of the calcaneofibular ligament.^11^ Studying different ligaments in the same joint can also reveal how the ligaments interact during an activity. A comparison of intact and ACL-deficient knees confirmed the importance of the ACL to knee stability by showing that ACL injury caused significant MCL extension and LCL shortening during lunges.^172^

The virtual fibre elongation method also provides insights into the strain distribution within tissues by using multiple fibres. This techniques has revealed that the anterior, middle, and posterior bundles of the superficial MCL exhibit different length change patterns (19%, 0%, and -16%) from full knee extension to 145° knee flexion during lunges.^71^ Similarly, the length of the anterior and posterior bundles of the LCL exhibited a 6-7% increase and a 10-16% decrease, respectively.^71^,^132^ Running induced significantly greater elongation of the ACL anteromedial bundle compared to the posterolateral bundle,^121^ whereas, walking showed more uniform elongation within the ACL.^181^ This approach also revealed that reconstructing the medial patellofemoral ligament (MPFL) to a location posterior and proximal to its anatomic femoral attachment results in the most isometric graft behaviour during lunging.^90^ Similarly, at the shoulder, virtual fibre elongation techniques have allowed an insight into otherwise difficult to access dynamic musculoskeletal interactions. Maximal external rotation at 90° abduction significantly elongates the anterior bundle of the inferior glenohumeral ligaments while only slightly elongating the posterior bundle. Conversely, maximum internal shoulder rotation at 90° abduction significantly elongates the posterior bundle while only slightly elongating the anterior bundle.^113^ Thus, the virtual fibre elongation method can reveal different function within regions of a single ligament, and consequently inform surgical reconstruction techniques and selective ligament release in joint revision surgery.

The virtual fibre elongation method can also evaluate the outcomes of surgical procedures via the length change patterns of ligaments or grafts during functional movements. A possible mechanism for poor outcomes in PCL-retaining total knee arthroplasty (TKA) patients was identified because PCL elongation showed a significant increase compared to healthy knees at flexion angles of over 75° during lunges.^189^ Cruciate-retaining TKA was also found to have a significant effect (mostly increasing) on the elongation patterns of the knee collateral ligaments during lunging by comparing the results of cruciate sacrificing TKA and healthy knees.^71^,^133^ A new study investigated the effect of knee TKA with ultra-congruent implants on the length change patterns of MCL and LCL during a variety of daily functional movements such as walking and squatting.^68^ In the TKA knees, while the LCL consistently slackened with increased knee flexion in all movements, the MCL showed regional variation in the length changes. The anterior, middle, posterior MCL fibres lengthened, remained isometric, and shortened during knee flexions, respectively. This confirmed the pervious observed distinct regional behaviour of the MCL^71^,^108^ and indicated that the location of partial MCL releases performed intraoperatively to balance the knee will have important consequences on post-operative stability. For ACL reconstruction, anatomic ACL graft placement better restores the length and length change patterns of the native ACL compared to an anteroproximal graft placement.^1^ A similar study found that compared to non-anatomic ACL reconstructions, anatomic ACL reconstruction results in PCL length change patterns (both bundles) that are closer to native patterns.^161^ Therefore, the virtual elongation method provides a unique opportunity to non-invasively evaluate native and graft soft tissue strains, hence enabling quantitative evaluation of surgical techniques.

### Advantages and Limitations

**Figure 4:**
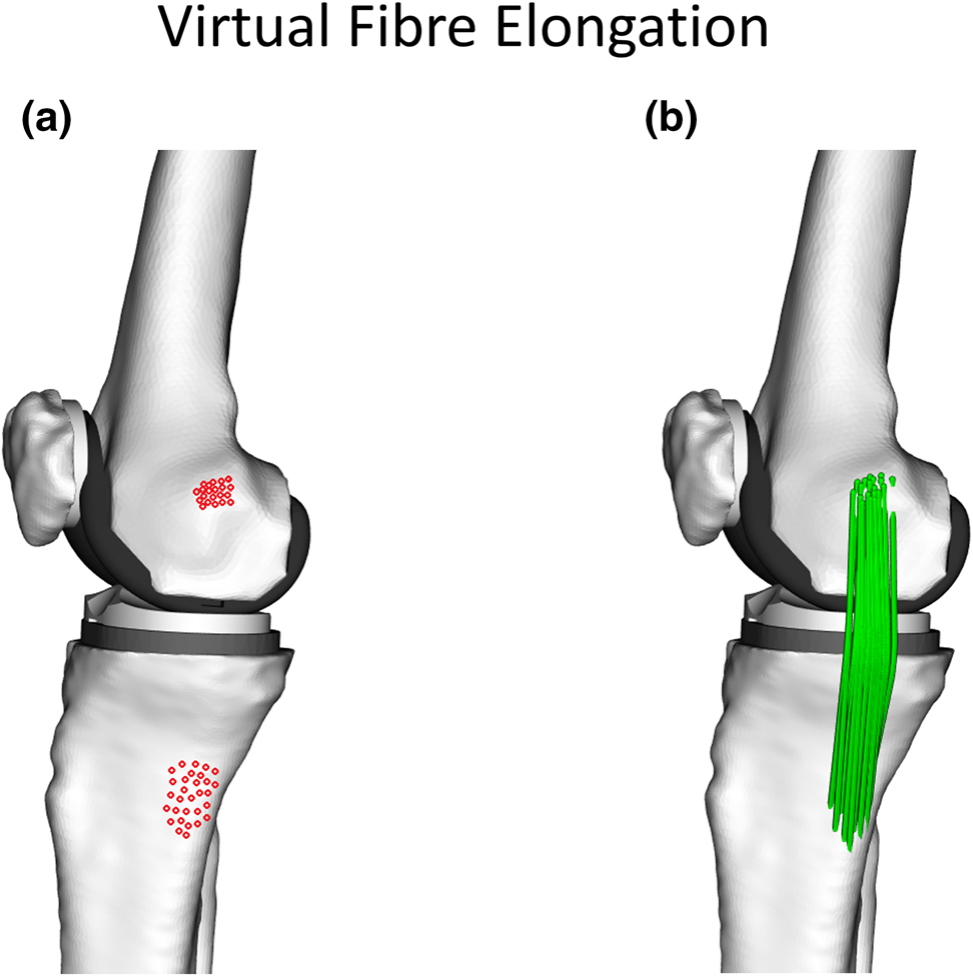
Strain measurements of the virtual fibres throughout dynamic activities enables the identification of sMCL isometric locations in the femoral and tibital attachment areas. (a) 32 femoral and tibial attachments on knee model of a TKA patient. (b) sMCL virtual fibres from different combinations of femoral and tibial attachments.

The virtual fibre elongation method leverages state-of-the-art dynamic imaging technologies to non-invasively evaluate soft tissue strains throughout functional activities at high frequencies (> 200 Hz). It can quantify regional tissue strains by using multiple fibres to represent the same ligament (Fig. 4). Furthermore, as the fibre attachment locations are manually selected, uncertainty quantification and sensitivity studies can be readily performed.^68^ Additionally, the measurement accuracy is not affected by whether the tissue is deep or superficial. Finally, the non-invasive nature of the method allows comparisons of injured or reconstructed tissue function against the healthy contralateral limb.

Despite the many advantages of the virtual elongation method, there are also several limitations:

1. Accuracy: Representing complex 3D tissue geometries with virtual fibre paths introduces several sources of error. Significant uncertainty in reported ligament lengths originates from both the identification of the tissue attachment footprints and virtual fibre insertion sites within the footprint.^90^ The determination of ligament insertion sites on MR images can show large variations especially in some ligaments like the sMCL, which has large attachment areas (nearly 400 mm^2^ on its tibial proximal attachment).^98^ However, for other ligaments such as the ACL, footprints can be accurately identified from MRI images.^10^ The simplification of ligament pathways to straight virtual fibre paths also affects the accuracy, as it neglects any variations in pathway length due to wrapping around skeletal structures. Furthermore, virtual fibre ligament elongations are also sensitive to the measurement accuracy of the bone kinematics. With longer fluoroscope shuttering times (fluoroscopic exposure time per frame), the X-ray image becomes blurred, leading to errors in the accuracy of the 2D–3D registration. As an example, Ellingson and co-workers reported that the errors in knee joint kinematics could be as large as 2.0° and 1.6 mm with an exposure time of 16 ms,^36^ which would propagate into errors in the assessment of virtual fibre elongation patterns. Finally, virtual fibres only provide bone-to-bone elongation measurements, which makes them well suited for ligaments, but they cannot assess muscle fibre lengths and tendon lengths independently.
2. Difficult to determine slack length: The resting length of ligaments cannot be measured using fluoroscopy. Therefore, while relative elongation patterns can be reported, true tissue strain patterns or loading conditions cannot be quantified. To circumvent this issue, many researchers define the reference length of a structure to be its length at e.g. full knee extension,^35^ or at an instant of the gait cycle e.g. heel strike.^70^ However, the lack of a consistent choice of reference length limits inter-study comparisons, and an appropriate solution should be agreed across the field.
3. Measurement frequency and radiation exposure: Fluoroscopes can generate either continuous 57 or pulsed 107 X-ray imaging. Continuous radiation modes enable higher measurement frequencies (speeds are then limited by the camera and fluoroscopic imaging formation), but also leads to greater radiation exposure to the subject, which constrains the measurement time and consequently the number of repetitions. Pulsed X-ray reduces the radiation dosage and therefore extends the measurement time. However, fluoroscopes with pulsed X-ray have limited measurement frequencies (often 25–30 Hz), which is insufficient to track highly dynamic movements such as impact situations.
4. Limited FOV: Traditional fluoroscopy systems are stationary with fields of view of 20-30 cm diameter. Thus, for many joints it is impossible to image complete activity cycles such as walking, running, or stair ascent/descent. Therefore, movements such as single legged lunge are often studied as the knee remains within the FOV.^90^,^173^ To track walking or running gait activities, the subject has to walk on a treadmill with only portions of the gait cycle such as stance phase being imaged.^108^,^181^

### Future Work

The virtual fibre elongation method has demonstrated great potential for assessing the function of ligaments in healthy^74^,^166^,^181^ and surgically reconstructed states^133^,^161^,^165^ in dynamic activities such as running^161^ and landing.^168^ However, the space constraints and stationary field of view of the imaging systems limit the movements that can be studied. Recently, mobile fluoroscopy systems have been developed to track the horizontal and vertical displacements of the joint during full body locomotion. Two of these systems have been developed to image lower limb joints throughout complete activity cycles.^59^,^107^ However, such systems may affect the subject’s gait performance and introduce additional errors due to vibrations.^66^

Currently, the virtual fibre elongation method is too time consuming for clinical translation due to the segmentation of the joint anatomy, definition of ligament attachment points, and 2D/3D images registration steps. Introducing artificial intelligence techniques to automate these processes could eliminate the time-consuming nature of this method, and therefore make it clinically viable. For example, a segmentation method combining image registration and machine learning has been developed to automatically segment the region of interest on ultrasound 185 and MRI images.^184^ It could be envisaged that such technology will help improve the automation of the virtual fibre elongation method. Moreover, instead of using virtual fibres to model the ligament, future study could develop the finite element model of the ligament and apply the joint kinematic data as boundary conditions to simulate the 3D strain-stress enviroment within the ligament.^117^ Finally, roentgen stereophotogrammetric analysis (RSA) can extend the virtual fibre elongation method to enable tendon strain measurement, especially to evaluate the recovery of the mechanical properties and function of reconstructed tissues.^2^,^86^,^147^,^148^ RSA requires that titanium beads are implanted in bones or soft tissues through open surgery^12^ or injection,^149^ and then uses dynamic imaging to capture the distance between beads throughout an activity to quantify tissue length changes. Tashman and co-workers have developed a dynamic RSA system by combining X-ray radiographs and high-speed digital imaging to achieve high measurement frequency (250 Hz) and accuracy (± 0.1 mm),^164^ which could be used in measuring dynamic activities. However, the invasive nature of bead implantation limits its application on human subjects.

## Ultrasound

Ultrasound is a non-invasive, radiation-free, and cost-effective technique to visualise the internal architecture of musculoskeletal soft tissues. Clinically, ultrasound is commonly used to identify pathological tendon abnormalities.^114^ In research settings, ultrasound has been leveraged to quantify muscle, tendon, and ligament deformation during dynamic activities. Numerous ultrasound studies quantified strains of large flat tendons such as the AT during walking 50,88 and running,^39^,^102^ however, very few ultrasound studies focused on ligaments.^155^ Ultrasound also enables muscle fibre lengths and pennation angle to be dynamically assessed.^33^,^97^

Typical ultrasound systems consist of a hand-held transducer connected by flexible wire to a cart that houses the electronics for signal generation and processing, as well as a screen for visualization. The working principle behind ultrasound imaging relies on propagating ultrasonic waves (sound waves with frequencies > 20,000 Hz) into the tissue of interest and recording their reflection.^139^,^146^ The ultrasonic waves reflect off the tissue according to the local properties, and the echoes are recorded and used to generate an image. Traditional ultrasound systems generate planar 2D images and can reach tissues up to 17 cm beneath the skin.^38^

**Figure 5:**
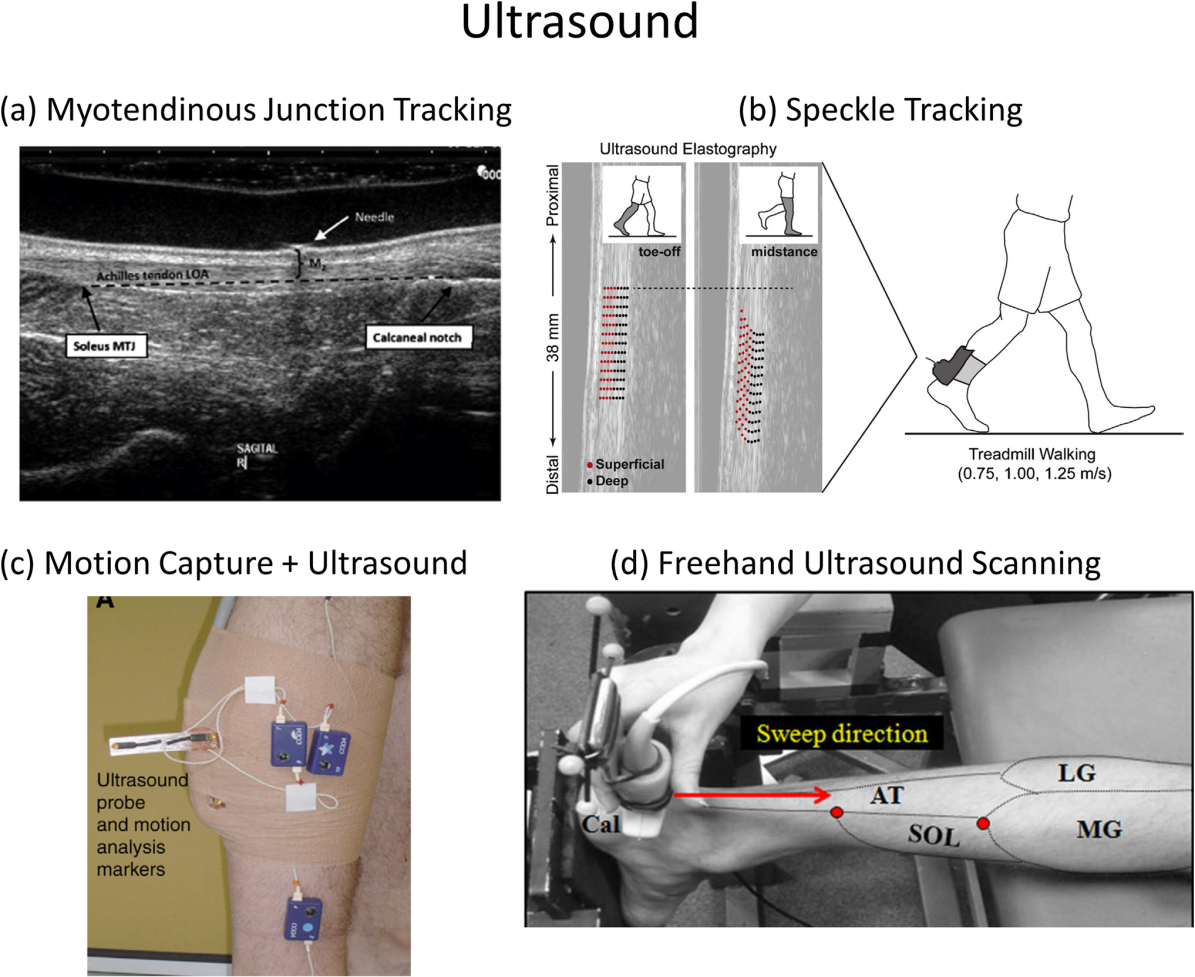
A variety of ultrasound techniques to quantify muscle and tendon elongations. (a) Ultrasound transducer with a wide FOV (100 mm) can image the entire free Achilles tendon (soleus to calcaneus). (b) The ultrasound elastography speckle tracking method enables the displacements of the superficial and deep AT tissues to independently measured during walking. (c) By fixing motion capture markers to the ultrasound transducer, tissue displacements in the sATMJ can be tracked in the laboratory coordinate system to quantify AT length changes. (d) An ultrasound transducer with markers attached is manually swept along the AT to take a series of ultrasound images of the AT. The ultrasound images and transducer positions are merged to reconstruct 3D geometries of the AT at multiple poses. (Included figures are adapted from Ref. 50, 94, 105, and 125).

Two primary techniques have been developed to measure soft tissue strain during dynamic movements using ultrasound, myotendinous junction (MJ) tracking (Fig. 5) and speckle tracking (Fig. 5). Freehand 3D ultrasound has also been applied to measure Achilles tendon strain, but only by imaging multiple static postures, and thus this review focuses on MJ and speckle tracking techniques.

### Measurement Technology and Experimental Methods

For both strain measurement techniques, the ultrasound transducers must be fixed to the body during dynamic movement to image a tissue. Typically, the ultrasound transducer is housed in a foam orthotic and strapped to the body using athletic tape.^50^ In many experiments, a treadmill is used so that the ultrasound cart can remain stationary. Commonly, markers are attached to the transducer and the ultrasound system is synchronized with a motion analysis system so that the transformation between the image field of view and other anatomic landmarks can be calculated (Fig. 5).

The MJ tracking technique enables muscle and tendon lengths to be quantified by tracking anatomical landmarks in each ultrasound image of a series collected throughout an activity. For example, the elongation of the free AT can be measured by tracking the displacement between the calcaneus attachment and the soleus-AT myotendinous junction (sATMJ).^94^ An ultrasound transducer with a wide FOV (100 mm) can image the entire free AT from the calcaneus to sATMJ^94^ (Fig. 5). For many applications and ultrasound systems, however, the tissue length exceeds the transducer’s FOV and thus only one attachment site can be tracked. Synchronizing the ultrasound system with an optic tracking system provides another means to track the length change of a long tendon during movement.^40^,^104^,^135^ To measure the AT, a reflective marker is placed on the calcaneus attachment to track its position. An ultrasound transducer equipped with reflective markers is fixed on the shank to image the position of the sATMJ, and then transform it into the global coordinate system. The free AT length is then defined as the distance between the positions of the calcaneus and the sATMJ.

Speckle tracking provides an alternative method to quantify regional tissue strain using ultrasound. A speckle is an interference pattern with bright and dark spots in B-mode or radiofrequency (RF) ultrasound images.^156^ The speckle pattern remains constant when the tissue structure is static and thus is thought to be representative of the tissue structure rather than measurement noise. Therefore, when the tissue microstructure is deformed, the speckle pattern changes accordingly.^22^ After the B-mode or RF ultrasound images of the tendon are obtained throughout an activity, a region of interest (ROI) containing the tendon structure is manually selected on one image.^95^ Then, automated speckle tracking algorithms are used to calculate the frame-to-frame speckle displacement within the ROI, which is then converted into tendon elongations.^50^,^134^

### Slack Length

Ultrasound provides two opportunities to assess the slack length of a tissue. Firstly, a tissue can be imaged at multiple joint postures until buckling can be identified in the B-mode images. This has been demonstrated on the patellar tendon.^151^,^154^ Secondly, a novel ultrasound imaging technique called shear wave elastography, which quantifies the speed of shear wave propagation in a tissue, has been applied to assess tissue slack length. Shear wave speed has been shown to be related to tissue stress,^23^,^111^ and thus can be applied to establish when the tissue goes slack if a corresponding joint is manipulated through its range of motion. This technique has been applied to assess the slack length of the AT^75^ and the ankle angle when the individual triceps surae muscles are slack.^65^

### Key Findings

Ultrasound based strain measurements have provided many important insights into the dynamic function of the AT. For example, ultrasound was used to quantify *in vivo* AT strains during dynamic activities such as running (3.5-5.8%), walking (4.6%), and one-legged hopping (8-8.3%).^40^,^41^,^104^,^105^ These functional AT strain data provide some important implications for AT injury. Firstly, the *in vivo* AT strains during one-legged hopping are surprisingly close to the AT failure strains found in *in vitro* tendon tensile tests.^15^,^104^ As the AT remained healthy after hopping tests, this finding demonstrates the difficulty of comparing *in vivo* and *in vitro* strain measurements and perhaps inconsistency among the methods. Secondly, the *in vivo* strain data from ultrasound measurements can serve as a variable to assess other parameters associated with AT injury, such as the mechanical “safe factor” 0 and “core temperature” 9 of the AT. Franz and co-workers measured the regional deformations of AT during walking,^50^ and showed that the aggregate AT exhibited nearly twice as much elongation as the free AT. Moreover, the superficial AT exhibited larger elongations than the deep AT and only the superficial AT elongation was found to increase at higher walking speeds, indicating non-uniform regional strain patterns in the AT. Cronin and co-workers compared the length changes of Achilles tendons during walking between healthy and diabetic subjects.^34^ The length changes of Achilles tendons were smaller in diabetic subjects than healthy patients but the length changes of muscle-tendon units were similar. These findings indicate that diabetes may decrease the elastic energy storage of Achilles tendons and increase muscle work, resulting in decreased walking efficiency. Finally, ultrasound has provided insights to AT healing and protection. For example, AT strain during running with a 18 mm heel lift was significantly smaller than barefoot, suggesting that such a heel lift could help to reduce the AT strains during AT rehabilitation training.^39^

Ultrasound evaluations have also contributed towards understanding the function of human triceps surae muscles during walking and running.^29^–^33^,^101^ In these studies, either a single ultrasound transducer was positioned over the medial gastrocnemius (MG) so that the soleus was also visible,^32^ or two transducers were placed over the MG and soleus.^80^ The MG and soleus fascicle lengths and pennation angles were then measured using an automated tracking algorithm, and motion-capture derived knee and ankle joint kinematics were further used to estimate MG and soleus muscle-tendon unit lengths.^32^,^102^,^116^ These studies found different length change patterns and magnitudes between the MG and soleus fascicles during the stance phase of gait, indicating the different functional roles of the two muscles.^80^ Relevant studies have also determined the differences in triceps surae length changes and behavior between walking and running,^81^,^102^ between forefoot and rearfoot running,^160^ and at different running speeds.^79^ Investigating the length change patterns in both muscle fascicle and muscle-tendon unit also showed a decreased energy storing role of the AT in race-walking compared to running.^32^ The effect of age on gastrocnemius muscle-tendon behavior during walking has also been studied by ultrasound,^116^ and it was found that tendon lengthening is greater and muscle fascicle lengthening is smaller in older subjects compared to young subjects. In addition, ultrasound has been used to characterize the effect body mass on gastrocnemius medialis fascicle behavior during stair ascent.^157^ By calculating the difference between the muscle tendon unit length change and the fascicle length change, it was found that additional body mass could cause substantially greater stretching on the tendon. This finding may be meaningful in evaluating effect of obesity on pathological loading in triceps surae muscle tendon unit.

The application of ultrasound to quantify ligament strain is limited to a few studies investigating superficial ligaments during static movements, such as the dorsal lisfranc ligament,^56^,^110^,^145^ pisohamate ligament and pisometacarpal ligament.^119^ Using speckle-tracking, 5-7% strains were observed in the coracoacromial ligament during dynamic shoulder rotations such as forward flexion, horizontal abduction, and internal rotation at 90° abduction.^134^ The strain patterns and displacements of the coracoacromial ligament indicated contact with the rotator cuff in these frequently used shoulder movements. This indicates a possible mechanism for rotator cuff pathologies, and provides guidelines for rehabilitation protocols as certain movements such as forward flexion, horizontal abduction, and internal rotation at 90° abduction were found to induce excessive tissue strains.

### Advantages and Limitations

Ultrasound provides several key advantages over other methods for assessing musculoskeletal soft tissue strain. Firstly, it is non-invasive, relatively inexpensive, and does not expose the subjects to radiation. Secondly, ultrasound can dynamically image soft tissues, which enables regional deformation within the tissue to be quantified. Finally, it enables the strains of muscle fibres and tendon to be independently measured as it can differentiate muscle-tendon interfaces.

Despite these advantages, ultrasound methods also suffer from several limitations:

1. Controversial reliability: Establishing good reliability of ultrasound tissue length measurement is fundamental to highly accurate measurements of tissue strain.^114^,^169^ However, the reliability of ultrasound measurement of static tissue geometry has been controversially reported,^5^,^14^,^13^,^54^,^78^,^120^,^150^ and these measurements are easier to perform than strain measurement during dynamic activities. Furthermore, transducer orientation and position have a significant effect on ultrasound measurements,^93^,^96^ and rigid fixation of the ultrasound transducer over the tissue of interest is difficult to achieve throughout dynamic activities. Some confidence can be gained from the good intra-class correlation coefficient (ICC) of ultrasound in measuring AT strains (0.72–0.86) and coefficient of multiple correlation of measuring PT strains (0.77–0.82),^22^,^150^ but it should be noted that these studies only measured tendon strain during static movements.
2. Challenging to measure ligament strains: Ultrasound is mostly used clinically to diagnose ligament injuries,^100^,^153^,^175^,^180^ but rarely to measure ligament strains. This is largely due to the difficulty of obtaining clear images of complete ligament structures as they are normally small and located beneath neighboring tissues. In addition, ligaments commonly are directly adjacent to bone and other hypoechoic tissues, which can lead to artefacts and reduced ligament visibility in ultrasound images. Furthermore, in traditional ultrasound images, the ligament appears hypoechoic at rest because of anisotropy artefacts and hyperechoic under loading due to tightening of the ligament microstructure.^30^,^119^ This change in appearance between loading conditions can help differentiate the ligament from other tissues but causes difficulties when trying to quantify ligament strain patterns throughout an entire movement.
3. Confounded by out-of-plane motion: The complexity of measuring the deformation of 3D soft tissues in planar 2D ultrasound images is another key limitation of ultrasound. MJ tracking and speckle tracking both require tracking landmarks or pixels in the 2D ultrasound image. Out-of-plane strains resulting from tissues bulging, rotating, or twisting may be missed or misinterpreted as in plane translation. Freehand 3D ultrasound addresses this limitation, but cannot be applied to dynamic movements because the transducer must be swept over the length of the tissue.^42^,^128^
4. Transducer fixation over tissue: For ultrasound measurement of dynamic activities, the transducer must be fixed over the tissue of interest as any motion of the transducer relative to the body can cause measurement error. This requires that the transducer is strapped to the body which may alter natural movement patterns. Finally, dynamic measurements are typically limited to a treadmill to avoid moving the ultrasound cart over-ground with the subject.

### Future work

Ultrasound methods are currently the most clinically viable for assessing musculoskeletal soft tissue strains due to their non-invasive, non-radiative, and relatively low-cost nature. However, their 2D nature limits the accuracy and repeatability of this method. Freehand 3D ultrasound scanning has been developed to quantify the 3D AT deformation *in vivo*,^42^,^125^ including changes in AT length, AT width, thickness, cross-sectional area, and volume.^125^,^127^ This technique relies on a conventional ultrasound machine coupled with a transducer instrumented with reflective markers and an optical tracking system. The operator performs transverse ultrasound scans from the calcaneus along the AT to the gastrocnemius muscle,^103^,^128^ during which the spatial position and orientation of the ultrasound transducer are recorded. Then, the 2D ultrasound images (between-frame interval of 0.1-0.5 mm) are transformed into the global coordinate system to create a 3D AT reconstruction.^129^ Highly precise 2D-to-3D transformation of the image (error under 1 mm) can be achieved after temporal and spatial calibration of the ultrasound transducer.^128^,^129^ Measurements of 3D AT deformations then require a series of digital processing procedures such as segmenting and rendering AT cross-sections, reconstructing 3D AT volumes, and defining measurement sites.^128^ The applicability of 3D freehand ultrasound to dynamic movements, however, remains to be established (Fig. 5).

## Summary and Perspectives

*In vivo* strain measurements have provided important insights into the mechanical function of musculoskeletal soft tissues, resulting in improved rehabilitative and surgical treatments in sports medicine. This review has presented three main state-of-the-art methodologies that enable quantification of soft tissue strain patterns during dynamic movements: implantable strain sensors, virtual fibre elongation, and ultrasound. Each methodology has provided key measurements that altered treatments and rehabilitation for musculoskeletal soft tissues. However, further innovations in the measurement technologies and their clinical implementation will be necessary to facilitate a data driven revolution to personalize sports medicine treatments.

Each strain measurement technique has its own virtues and limitations in terms of safety, application, and accuracy (Table 1). Implantable sensors enable strains in deep tissues to be measured with high accuracy and frequency. However, the sensor designs that have been implanted in humans are highly invasive, can impinge on neighbouring tissues, limit the movements that can be performed, and require a data transmission wire to cross the skin. The virtual fibre elongation method enables elongation measurements of multiple deep and superficial tissues during highly dynamic movements. However, it can expose the subjects to radiation depending on the imaging method, and can only quantify bone-to-bone tissue length changes and thus cannot differentiate between muscle and tendon strain. Ultrasound methods provide unique advantages because they are non-invasive, do not expose subjects to radiation, and provide direct imaging of the soft tissue structures. Ultrasound also provides the unique possibility to measure regional tissue strains compared to implantable sensors and the virtual fibre elongation methods which can only assess point-to-point strains. However, dynamic ultrasound has limited accuracy due to out-of-plane motion, and is largely only applicable to measure superficial muscles and tendons. Thus, researchers and clinicians must carefully consider the advantages and limitations of each measurement technique when planning new studies and interpreting results.

**Table 1:** Functional characteristics of each strain measurement method.

Measurement method	Measurement mode	Radiation	Accessibility	Measurement area	Dynamic activities measured	**Clinical application**
Strain sensor	Invasive	No	ACL	Anteromedial bundle Regional strain	Squat Bicycle Step up/down* lunge	Intraoperative function assessment of ACL graft Long-term follow-up function change of ACL graft
Virtual fibre elongation (with fluoroscope)	Non-invasive	Yes	Knee joint: ACL PCL LCL MCL MPFL Ankle joint: ATFL CFL Shoulder joint: glenohumeral ligaments	Multiple bundles Overall strain	Walk Run Hop Jump land Step up Lunge	Function assessment of knee ligaments after total knee arthroplasty Identification of tunnel placement effect on graft function
Ultrasound	Non-invasive	No	AT PT Limited superficial ligaments	3D Regional & overall strain	Walk Run Hop	Investigation of tendinopathy effect on tendon mechanical properties Investigation of tendon adaptation to rehabilitation therapy

*Including similar movements such as standing up from sitting posture

Assessment of soft tissue slack length remains significantly challenging for each strain measurement technique. The inflection point method has been well validated for determining ACL slack length with implantable strain sensors, but it is uncertain whether similar experiments can be replicated on other soft tissues. The virtual fibre method currently has no reported method for measuring slack length, but potentially could be coupled with ultrasound. Ultrasound can provide measurements of slack length through visualizing tissue buckling or using shear wave propagation,^112^ but these may not be applicable to all tissues. Future studies should at the very least report the method used to determine the reference length and provide quantitative values of the tissue lengths.

A key limitation of this study is that it only focused on measurement techniques that have been applied to measure *in vivo* human musculoskeletal soft tissue strains during functional movements. However, several techniques have been used to measure *in vivo* strains in animal tissue (sonomicrometry,^118^ liquid metal strain gauges^26^), *in vivo* strains in human bone,^49^ and *in vivo* loading in human tendons (fibre optic sensors,^43^ buckle transducers,^52^ shear wave tensiometers^111^). Furthermore, novel techniques such as near infrared spectroscopy^170^ show promise to quantify musculoskeletal soft tissue mechanical properties. The working principles behind any of these techniques may lead to the needed breakthrough that overcomes the limitations of current strain measurement methods.

In conclusion, we have found *in vivo* strain measurements have improved clinical treatments for many musculoskeletal pathologies including ACL reconstruction, AT repair, and TKA. However, a new generation of clinically viable technologies are needed to facilitate a data driven progression of personalized surgical and rehabilitative sports medicine treatments.

## Abbreviations

ACL: Anterior cruciate ligament
AT: Achilles tendon
MRI: Magnetic resonance imaging
CT: Computed tomography
HEST: Hall Effect Strain Transducer
DVRT: Differential Variable Reluctance Transducer
FOV: Field of view
sMCL: Superficial medial collateral ligament
MPFL: Medial patellofemoral ligament
TKA: Total knee arthroplasty
RSA: Roentgen stereophotogrammetric analysis
MJ: Myotendinous junction
sATMJ: Soleus-AT myotendinous junction
RF: Radiofrequency
ROI: Region of interest
MG: Medial gastrocnemius
ICC: Intra-class correlation coefficient
